# Role of fibroblast growth factor 23 in predicting CKD progression and prognosis: a systematic review and meta-analysis

**DOI:** 10.7717/peerj.21668

**Published:** 2026-08-24

**Authors:** Jia-Wei Hsu, Hsiang-Ping Wang, Tsay-I Chiang

**Affiliations:** 1 Division of Nephrology, Taichung Veterans General Hospital, Taichung City, Taiwan; 2 Department of Nursing, HungKuang University, Taichung City, Taiwan

**Keywords:** Fibroblast growth factor 23 (FGF-23), Meta-analysis, Chronic kidney disease (CKD), Biomarker

## Abstract

**Background and Objectives:**

Chronic kidney disease (CKD) affects 10% of the global population. This meta-analysis evaluated fibroblast growth factor 23 (FGF-23) as a prognostic biomarker for CKD progression, kidney failure, cardiovascular events, and mortality.

**Materials and Methods:**

We systematically searched PubMed, Cochrane, Embase, and SCOPUS from inception through January 2025. Twelve prospective cohort studies enrolling 21,042 pre-dialysis adult patients with CKD stages 1–5 were included; outcomes encompassed kidney function decline, progression to kidney failure, cardiovascular events, heart failure, and all-cause mortality.

**Results:**

Elevated fibroblast growth factor (FGF)-23 levels were associated with lower estimated glomerular filtration rate (eGFR) (mean difference: 17.83 mL/min/1.73m^2^). In mild-moderate CKD, each 10 relative units (RU)/mL FGF-23 increase conferred 1.7% higher kidney failure risk (hazard ratio (HR) 1.01, 95% confidence interval (CI) [1.01–1.02]). In advanced CKD, highest *vs*. lowest FGF-23 quartile showed 2.32-fold increased kidney failure risk (95% CI [1.80–2.98]). High FGF-23 associated with increased cardiovascular events (HR 1.72, 95% CI [1.34–2.20]), heart failure (HR 2.14, 95% CI [1.53–3.00]), and all-cause mortality (HR 1.61, 95% CI [1.40–1.87]).

**Conclusions:**

FGF-23 is a significant prognostic biomarker predicting CKD progression, cardiovascular events, and mortality, supporting its utility in risk stratification and personalized management. Incorporating FGF-23 measurement into routine CKD care may enable earlier risk stratification and more targeted clinical interventions.

## Introduction

Chronic kidney disease (CKD) affects an estimated 10% of the adult population globally, although this rate may partly reflect age-related decline in estimated glomerular filtration rate (eGFR) rather than true pathological kidney disease (GBD Chronic Kidney Disease Collaboration, 2020; Francis et al., 2024). The progressive nature of CKD highlights the urgent need for precise prognostic tools to guide clinical decision-making and enhance patient outcomes. While conventional markers such as eGFR and albuminuria remain essential for assessment, there is increasing interest in novel biomarkers that may provide additional prognostic insights (Kidney Disease: Improving Global Outcomes (KDIGO), CKD Work Group, 2024).

This meta-analysis investigates fibroblast growth factor 23 (FGF-23) as a promising biomarker in CKD. The selection of FGF-23 is based on its biological relevance, emerging evidence, and potential to provide complementary information to existing prognostic factors in CKD. FGF-23, a hormone produced by bones, plays a key role in regulating phosphate and vitamin D metabolism and has been recognized as a significant predictor of adverse outcomes in CKD (Mace, Olgaard & Lewin, 2020; Kurpas et al., 2021). Elevated FGF-23 levels correlate with CKD progression (Fliser et al., 2007; Lundberg et al., 2012; Tripepi et al., 2015; De Jong et al., 2021), increased cardiovascular events (Kendrick et al., 2011; Ix et al., 2012; Nakano et al., 2012; Bouma-de Krijger et al., 2014; Scialla et al., 2014; Seiler et al., 2014; Alderson et al., 2021), and elevated mortality risk (Isakova et al., 2011a; Kendrick et al., 2011; Ix et al., 2012; Nakano et al., 2012; Bouma-de Krijger et al., 2014; Levin et al., 2014; Seiler et al., 2014; Isakova et al., 2018; Alderson et al., 2021; De Jong et al., 2021). Notably, FGF-23 levels can rise early in CKD, often preceding changes in serum phosphate or parathyroid hormone (Isakova et al., 2011a), thereby positioning it as a potential early marker for mineral bone disorder and cardiovascular risk in CKD patients.

The primary objective of this systematic review and meta-analysis is to evaluate the prognostic value of serum FGF-23 in pre-dialysis adult patients with CKD stages 1–5. Specifically, we address the following question: among adults with established CKD not yet requiring kidney replacement therapy (KRT), are elevated baseline FGF-23 levels independently associated with an increased risk of kidney function decline, progression to kidney failure, cardiovascular events, heart failure hospitalization, and all-cause mortality? By synthesizing evidence from prospective cohort studies, this meta-analysis aims to provide robust pooled effect estimates and inform the utility of FGF-23 in clinical risk stratification and CKD management.

## Materials and Methods

### Search strategy and study selection

Search terms were developed using the Population, Exposure, Comparator, and Outcome (PECO) framework. The population was defined as adults (≥18 years) with confirmed CKD stages 1–5 not requiring KRT at baseline; the exposure was baseline serum FGF-23 measurement using C-terminal or intact assays; the comparator was low *vs*. high FGF-23 levels as defined within each study; and the outcomes were kidney function decline, progression to kidney failure, cardiovascular events, heart failure hospitalization, and all-cause mortality. MeSH terms and keywords corresponding to each PECO element were combined using Boolean operators (AND, OR) to construct the search strings applied across PubMed, Cochrane Library, Embase, and SCOPUS.

**Figure 1:**
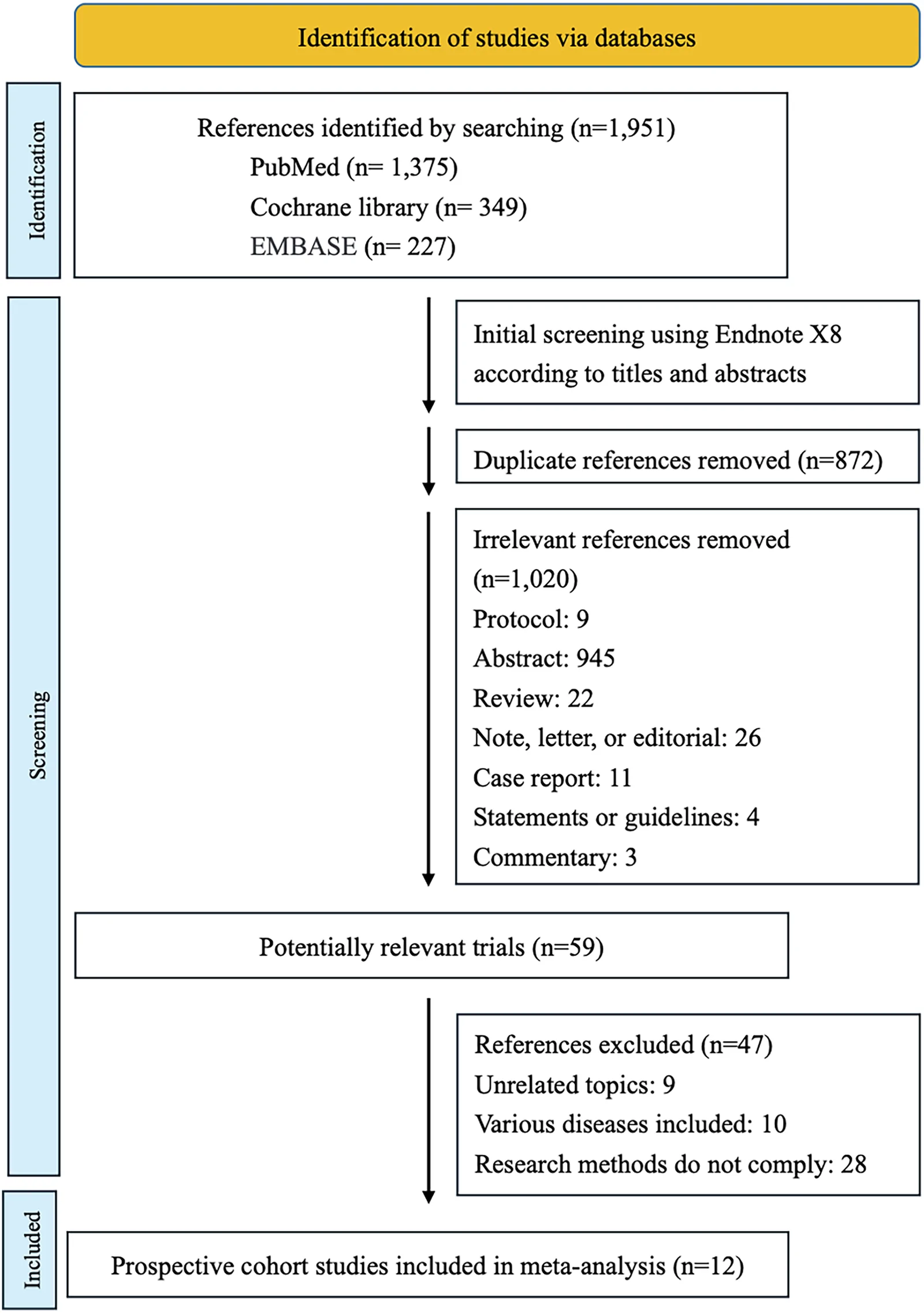
Study flow diagram demonstrating the article selection process for the meta-analysis.

We conducted a comprehensive systematic literature search across PubMed, Cochrane Library, Embase, and SCOPUS from inception through January 2025. No language or country restrictions were applied. The search strategy included, but was not limited to, terms related to chronic kidney disease across all stages, FGF-23, cardiovascular disease, kidney failure, and all-cause mortality, with a broad range of synonymous and related terminology also incorporated to maximize retrieval sensitivity. An updated literature search was conducted in February 2026 using the identical search strategy to identify studies published between January 2025 and submission; this search identified 13 additional records, none of which met our inclusion criteria. The complete study selection process, including records identified, screened, assessed for eligibility, and included, is summarized in the PRISMA flow diagram (Fig. 1).

### Inclusion and exclusion criteria

The current study included prospective cohort studies measuring FGF-23 levels in patients with CKD. The patients of the included trials were adults (≥18 years) with confirmed CKD stages 1–5 (not on dialysis). CKD was defined according to the Kidney Disease: Improving Global Outcomes (KDIGO) 2024 Clinical Practice Guideline criteria. The minimum follow-up duration was 12 months. Studies were excluded if they: (1) did not clearly report patient inclusion and exclusion criteria; (2) focused exclusively on acute kidney injury or kidney transplant recipients; (3) enrolled patients receiving KRT at baseline; (4) did not report baseline FGF-23 measurements; (5) employed unclear or insufficiently described FGF-23 measurement methods; (6) lacked extractable outcomes or sufficient data for effect size calculation; (7) were case reports, narrative reviews, editorials, or conference abstracts; or (8) represented duplicate publications of the same study cohort.

### Outcomes

Our systematic review and meta-analysis included studies that evaluated at least one of five key outcomes. The primary outcomes of interest were: (1) CKD progression rate, defined as advancement to a higher CKD stage or a predefined percentage decline in eGFR; (2) incidence of kidney failure, defined as initiation of chronic dialysis (hemodialysis or peritoneal dialysis), kidney transplantation, or eGFR declining to <15 mL/min/1.73m^2^ for >3 months. We included studies using various kidney failure definitions given the clinical reality that dialysis initiation timing may vary across healthcare systems and patient preferences, but all represent clinically relevant endpoints indicating kidney failure; (3) cardiovascular disease (CVD) events, defined as composite endpoints including fatal or non-fatal myocardial infarction, stroke, hospitalization for unstable angina, coronary revascularization procedures, or cardiovascular death. We recognize that CVD definitions varied across studies, with some using broader composite endpoints while others focused on specific cardiovascular outcomes. Studies were required to use standardized, adjudicated CVD event definitions based on established criteria (*e.g*., International Classification of Diseases codes); (4) heart failure events, defined as hospitalization for acute decompensated heart failure or heart failure-related death. Heart failure diagnoses were based on clinical criteria, administrative codes (ICD-9/ICD-10), or physician adjudication. We included studies examining incident heart failure as well as heart failure hospitalizations in broader CKD populations. Some studies further stratified by heart failure subtype, though the primary analysis combined all heart failure events; (5) All-cause mortality, defined as death from any cause during follow-up. We extracted outcome definitions exactly as reported in each individual study.

Although our initial literature search included bone and mineral disorder in CKD as a potential outcome of interest, we found that none of the studies meeting our inclusion criteria evaluated this as a primary outcome. Therefore, this outcome was not included in our final analysis, which focused exclusively on the five clinically relevant endpoints listed above that were consistently reported across the selected studies.

### Data extraction and methodological quality appraisal

Records from PubMed and the Cochrane Library were managed using Endnote X8 (Clarivate Analytics), with screening conducted based on title and abstract, and full-text reports were obtained for further evaluation. Two reviewers (Jia-Wei Hsu, Tsay-I Chiang) independently conducted parallel literature searches to minimize selection bias and extracted the reference information: first author, study design, publication year, countries, study size, gender, average age, follow-up duration, FGF23 measurement, FGF23 categories, lower and upper range of the FGF23 assay, reference levels, CKD stages, kidney functions, and outcome data from the included trials. Disagreements were resolved by a third reviewer (Hsiang-Ping Wang). The same reviewers evaluated the methodological quality based on Newcastle-Ottawa scale (NOS) for non-randomized studies regarding the selection (0–4 points), and the comparability (0–2 points) of study groups and the ascertainment of the interested outcome (0–3 points) independently (Wells et al., 2014). The disagreement was discussed and consulted with the same third reviewer for the final judgment.

Among the 12 included studies, 10 used C-terminal FGF-23 (cFGF-23) assays (predominantly second-generation Immutopics enzyme-linked immunosorbent assay (ELISA), one used intact FGF-23 (iFGF-23) assays (Kainos), and one study measured both forms). These assay types differ fundamentally in what they measure: cFGF-23 assays detect both the intact hormone and its C-terminal cleavage fragments, while iFGF-23 assays specifically measure only the biologically active, full-length FGF-23 molecule. This methodological difference has important implications, as cFGF-23 levels are typically 5–10 fold higher than iFGF-23 levels in the same sample due to inclusion of degradation products. To account for this methodological heterogeneity, we carefully documented the assay type, manufacturer, and units of measurement for each study (Table 1).

**Table 1:** Characteristics of included cohort trials.

Author, year	Design	Location	Sample size, *n*	CKD stage	Median follow-up years	FGF23 category	FGF23 measurement (IQR)^a^	FGF23 assay (brand name)	eGFR, mean ± SD (ml/min/1.73m^2^)^a^	Measurement formula of eGFR	Risk outcomes
Isakova et al. (2011b)	PC	USA	3,879	2–4	3.5	<95.8, 95.8–145.4, 145.5–239.1, >239.2 RU/mL	cFGF23 145.5 (96–239) RU/mL	ELISA (Immutopics)	42.8 ± 13.5	MDRD	ESRD, All-cause mortality
Kendrick et al. (2011)	PC	USA	1,099	3–4	2.9	<216, 217–380, 381–945, >946 RU/mL	cFGF23 392 (216–945) RU/mL	ELISA (Immutopics)	18 ± 6	MDRD	ESRD, CVD events, All-cause mortality
Scialla et al. (2014)	PC	USA	3,860	2–4	3.7	<96, 96–145.4, 145.4–238.9, >239 RU/mL	cFGF23 145.4 (96–238.8) RU/mL	ELISA (Immutopics)	44 ± 15	CKD-EPI and ^125^I-iothalamate	CVD events
Fliser et al. (2007)	PC	Germany, Austria, and Italy	177	1–5	4.4	<104, >104 RU/mL; <35, >35 pg/mL	cFGF23 222 (41–492) RU/mL; iFGF23 52 (18–80) pg/mL	cFGF23 and iFGF23 ELISA (Immutopics)	64 ± 38	Iohexol clearance	Renal events
Alderson et al. (2021)	PC	UK	388	3–5	5.8	Four quartiles of delta cFGF23	cFGF23 162 (101–244) RU/mL	cFGF23 ELISA (Immutopic)	NA	MDRD	ESRD, CVD events, All-cause mortality
Isakova et al. (2018)	PC	USA	1,135	2–4	5	Stable (80.2–139.9), slowly rising (159.8–304.0), rapidly rising (334.8–749.1)	cFGF23 145.0 (96.0–231.8) RU/mL	cFGF23 ELISA (Immutopic)	40 ± 17.4	MDRD	All-cause mortality
Levin et al. (2014)	PC	Canada	2,544	3–4	3	NA	cFGF23 237 (150–432) RU/mL	cFGF23 ELISA (Immutopic)	27.9 ± 9	MDRD	All-cause mortality, RRT or dialysis
Ix et al. (2012)	PC	USA	3,107	1–5	10.5	<51, 51–70, 71–100, >100 RU/mL	cFGF23 70 (53–99) RU/mL	cFGF23 ELISA (Immutopic)	51 ± 21	CKD-EPI	All-cause mortality, CVD events, HF
Tripepi et al. (2015)	PC	Italy	758	2–5	3	<41.4, 41.4–60.7, 60.7–91.2, >91.2 pg/mL	iFGF23 60.7 (41.4–91.2) pg/mL	iFGF23 ELISA (Kainos)	36 ± 13	MDRD	Renal events
Seiler et al. (2014)	PC	Germany	444	2–4	2.6	NA	cFGF23 102 (64–164) RU/mL	cFGF23 ELISA (Immutopic)	45 ± 16	MDRD	All-cause mortality, CVD events, HF
Leidner et al. (2023)	PC	USA	3,502	2–4	10.8	14–93.5, 93.6–138.7, 138.8–224.3, 224.5–14,318.9 RU/mL	cFGF23 138.7 (93.5–224.3) RU/mL	cFGF23 ELISA (Immutopic)	45 ± 15.4	MDRD	HF
Seiler et al. (2010)	PC	Germany	149	2–4	4.8	>104, ≤104 RU/mL	cFGF23 95 (16–759) RU/mL	cFGF23 ELISA (Immutopic)	36 ± 23	MDRD	CVD events

**Notes:**

IQR, interquartile range; PC, prospective cohort studies; ELISA, enzyme linked immunosorbent assay; eGFR, estimated glomerular filtration rate; HF, heart failure; CVD, cardiovascular diseases; cFGF23, C-terminal FGF23; iFGF23, intact FGF23; ESRD, end-stage renal disease; MDRD, Modification of Diet in Renal Disease equation; CKD-EPI, CKD Epidemiology Collaboration equation; RRT, renal replacement therapy; NA, not available.

a Data are presented as the mean ± SD or the median and IQR, as appropriate.

### FGF-23 assay types and measurement considerations

FGF23 exists in circulation in two main forms: the intact, biologically active full-length hormone and inactive C-terminal fragments. cFGF-23 assays detect both the intact hormone and C-terminal cleavage fragments. The most commonly used cFGF-23 assay is the second-generation Immutopics ELISA, which reports results in relative units (RU/mL). iFGF-23 assays specifically measuring only the full-length, biologically active hormone. The most commonly used iFGF-23 assay is manufactured by Kainos Laboratories (Tokyo, Japan), reporting results in pg/mL. In this meta-analysis, we included studies using either assay type but carefully tracked this methodological characteristic. Because the assays measure different analytes with different units, we did not pool absolute FGF-23 concentrations across assay types. Instead, our analyses used relative comparisons within each study (*e.g*., highest *vs*. lowest quartile, or standardized per-SD increases), which allows meaningful synthesis across different measurement methods.

### Heterogeneity

We evaluated heterogeneity using Cochrane Q tests and I2 statistics. I2 values of less than 25% were considered to indicate mild heterogeneity, 25–50% moderate heterogeneity, 50–75% substantial heterogeneity, and values above 75% considerable heterogeneity. To explore potential sources of heterogeneity, we conducted pre-specified subgroup analyses when I^2^ > 50% and >3 studies were available. Subgroup analyses examined: (1) baseline kidney function (eGFR <30 *vs*. 30–60 *vs*. >60 mL/min/1.73m^2^), (2) FGF-23 assay type (C-terminal *vs*. intact FGF-23), (3) follow-up duration (<5 *vs*. >5 years), (4) study population characteristics, and (5) geographic region. Meta-regression was performed to quantitatively assess the relationship between continuous moderators (mean baseline eGFR, median follow-up duration) and effect size when >10 studies were available.

### Statistical analysis

Data analyses were performed using RevMan version 5.4 (The Cochrane Collaboration, Oxford, England), and metaanalysisonline.com. Given anticipated between-study heterogeneity, a random-effects model was applied to all primary analyses. For categorical outcomes, hazard ratios (HR) were calculated to estimate effect sizes; mean differences (MD) were used for continuous outcomes. All effect estimates are presented with 95% confidence intervals (95% CI); statistical significance was set at *p* < 0.05. Between-study heterogeneity was quantified using the Cochran Q statistic and I^2^ statistic, with I^2^ values of <25%, 25–50%, 50–75%, and >75% interpreted as mild, moderate, substantial, and considerable heterogeneity, respectively.

This review was conducted in accordance with the Preferred Reporting Items for Systematic Reviews and Meta-Analyses (PRISMA) guidelines (Moher et al., 2009) and was prospectively registered in PROSPERO International Prospective Register of Systematic Reviews prior to data extraction (registration number CRD42024613649).

## Results

### Selection of studies

Figure 1 illustrates the PRISMA flowchart outlining the systematic literature search and study selection process. Our comprehensive database search identified 1,951 records across three databases: 1,375 from PubMed, 349 from Cochrane Library, and 227 from Embase. After removing 872 duplicate records, 1,079 unique records underwent title and abstract screening. Subsequently, 59 reports were assessed for full-text eligibility, with 47 excluded for not meeting the predetermined inclusion criteria. Finally, 12 studies were included in the comprehensive meta-analysis (Fliser et al., 2007; Seiler et al., 2010; Isakova et al., 2011a, 2011b; Kendrick et al., 2011; Ix et al., 2012; Levin et al., 2014; Scialla et al., 2014; Seiler et al., 2014; Tripepi et al., 2015; Isakova et al., 2018; Alderson et al., 2021; Leidner et al., 2023).

The 12 included prospective cohort studies enrolled 21,042 pre-dialysis adult patients with CKD stages 1–5 (sample sizes 149–3,879), conducted across the United States (*n* = 6), Europe (*n* = 5), and Canada (*n* = 1), and published between 2007 and 2023. Baseline eGFR ranged from a mean of 18 to 64 mL/min/1.73m^2^, reflecting varying CKD severity across cohorts. FGF-23 was measured using C-terminal assays in 10 studies, intact assays in one, and both forms in one. Outcomes examined included eGFR decline, progression to kidney failure, cardiovascular events, heart failure hospitalization, and all-cause mortality, with individual studies reporting one to three endpoints. Full study characteristics are presented in Table 1.

### Critical appraisal of the included studies

Table 2 presents the NOS quality assessment scores for the prospective studies included. All twelve included studies were prospective and demonstrated good methodological quality, with total NOS scores ranging between seven and nine points (Table 2). Notably, no studies were identified as having a high risk of bias related to blinding. The consistent scoring across studies suggests robust methodological quality, which enhance the credibility of the systematic review and meta-analysis.

**Table 2:** The newcastle-ottawa scale (NOS) scores.

Author, year	Study design	NOS score		
		Selection	Comparability	Outcome
Isakova et al. (2011b)	Prospective	4	1	3
Kendrick et al. (2011)	Prospective	4	1	3
Scialla et al. (2014)	Prospective	4	1	3
Fliser et al. (2007)	Prospective	4	1	3
Alderson et al. (2021)	Prospective	3	1	3
Isakova et al. (2018)	Prospective	4	2	3
Levin et al. (2014)	Prospective	3	1	3
Ix et al. (2012)	Prospective	4	1	3
Tripepi et al. (2015)	Prospective	4	1	3
Seiler et al. (2014)	Prospective	3	1	3
Leidner et al. (2023)	Prospective	4	2	3
Seiler et al. (2010)	Prospective	4	1	3

**Note:**

The NOS score is an aggregate score derived from three distinct domains: Selection (with a maximum of four points), Comparability (with a maximum of two points), and Outcome (with a maximum of three points). NOS, Newcastle-Ottawa Scale.

### Association between FGF-23 levels and CKD stage

**Figure 2:**
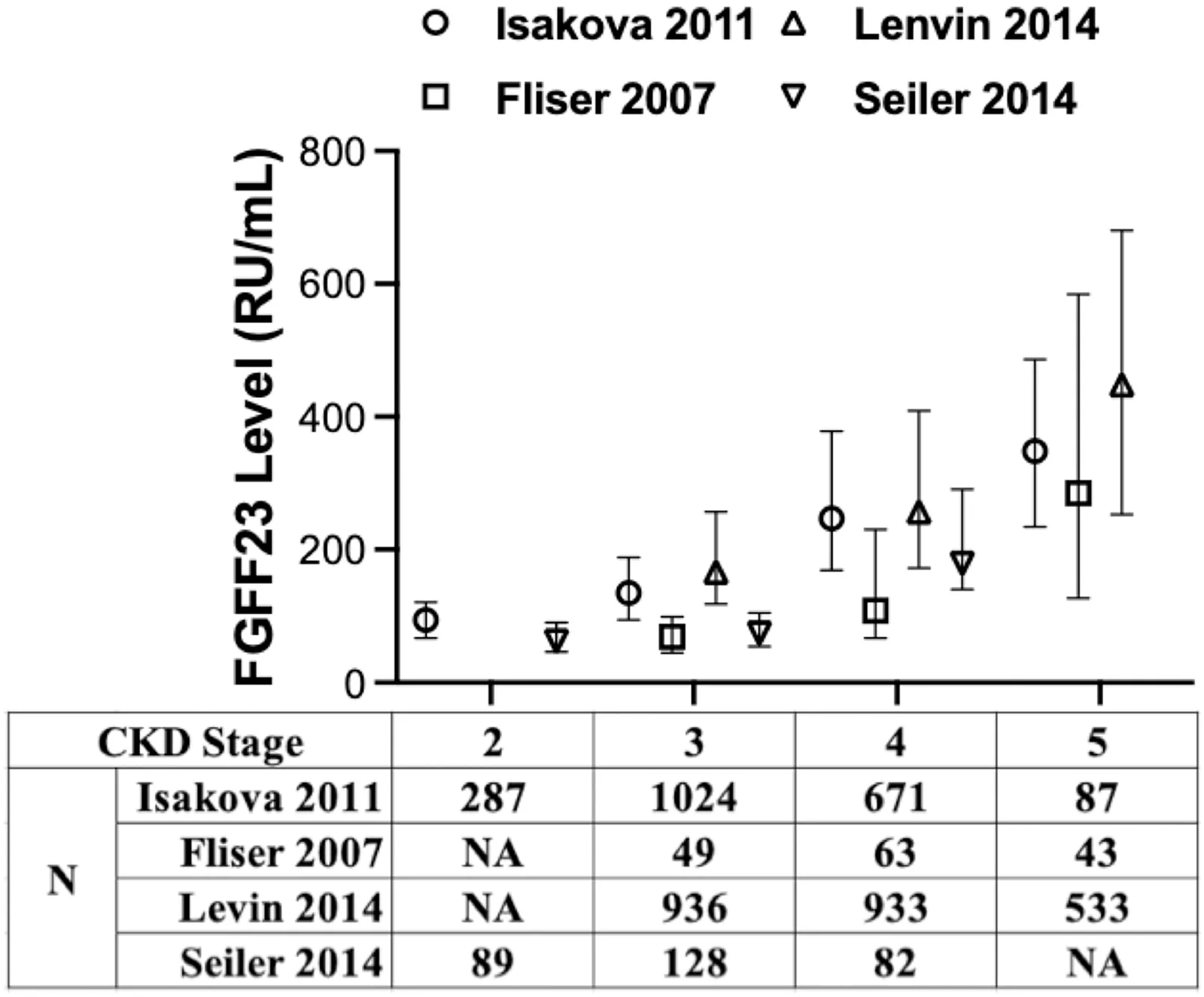
Increase in serum FGF23 levels with advanced CKD stage. Median FGF23 levels (RU/mL) for each CKD stage across four prospective cohort studies. The y-axis represents the FGF23 level, while the x-axis displays the CKD stages, ranging from stage 2 to stage 5. Bars and symbols represent the interquartile range and median value, respectively.

Understanding the relationship between FGF-23 and chronic kidney disease severity is crucial for interpreting its potential role as a biomarker. We examined the cross-sectional association between FGF-23 levels and CKD stage across four prospective cohort studies (Fliser et al., 2007; Isakova et al., 2011a; Levin et al., 2014; Seiler et al., 2014). As demonstrated in Fig. 2, a clear trend of increasing FGF-23 levels was observed with advancing CKD stages. For example, in the Isakova 2011 study, median FGF-23 levels rose from 94 RU/mL in stage 2 CKD to 135 RU/mL in stage 3, 247 RU/mL in stage 4, and 348 RU/mL in stage 5 CKD (Fig. 2). Similar patterns were consistently reported across the other included studies, demonstrating a strong cross-sectional relationship between FGF-23 levels and kidney function.

These findings demonstrate a strong association between FGF-23 levels and CKD stage, with FGF-23 concentrations progressively increasing as kidney function declines.

To address whether FGF-23 independently predicts CKD progression, longitudinal analyses from several included studies provide additional insights. Fliser et al. (2007) reported that baseline FGF-23 levels predicted future decline in kidney function even after adjustment for baseline eGFR and other confounders. Similarly, Isakova et al. (2011a) found that higher baseline FGF-23 was associated with greater risk of progression to more advanced CKD stages independent of baseline kidney function. These findings suggest that FGF-23 may provide prognostic information beyond serving as a marker of current CKD severity.

### Association between elevated serum FGF23 levels and decreased eGFR in CKD

**Figure 3:**
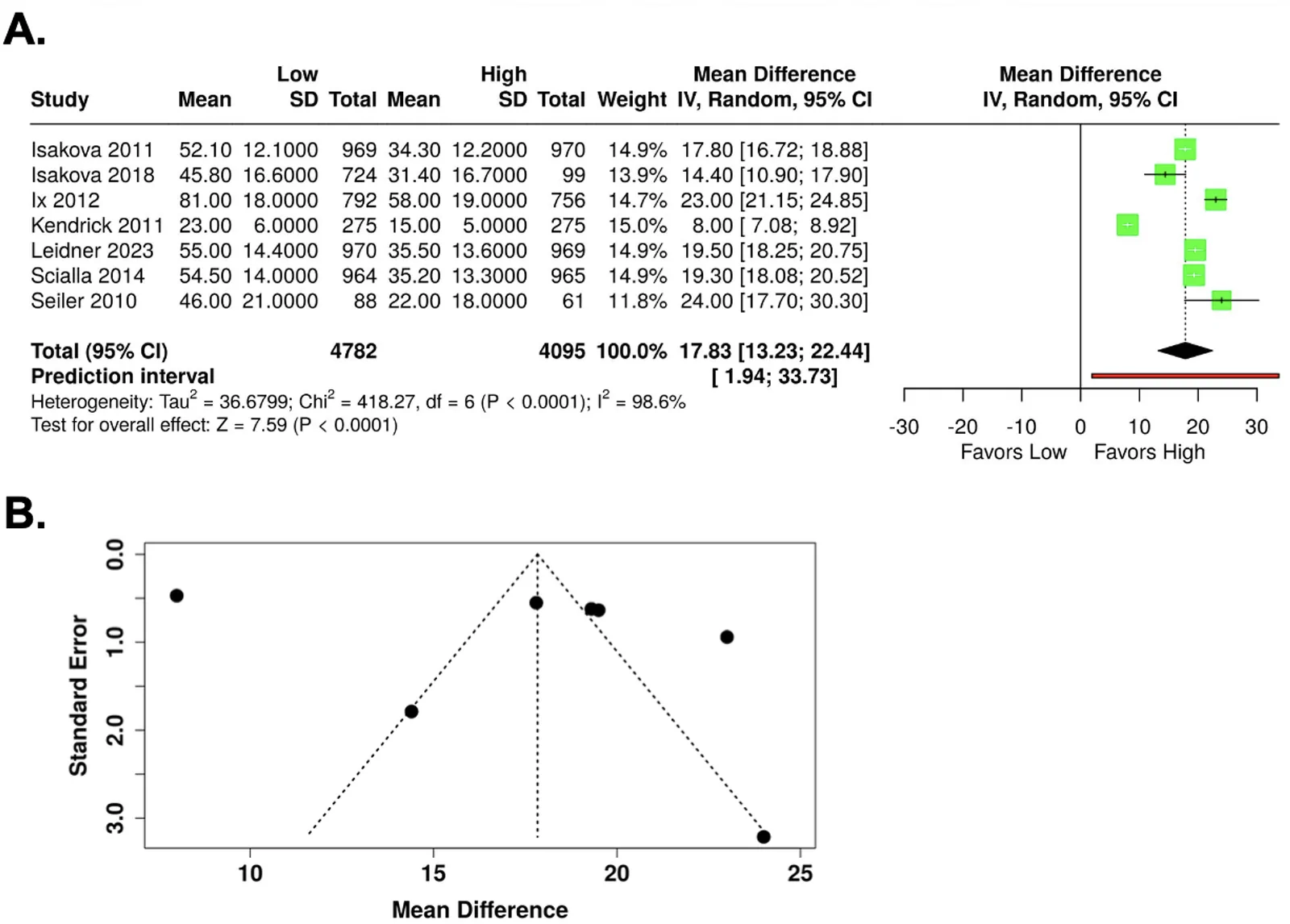
Association between serum FGF23 levels and eGFR in CKD. (A) The mean difference in eGFR (mL/min/1.73m^2^) between participants with low *vs*. high FGF23 levels from individual studies and the pooled estimate. FGF23 levels are categorized into Low (Q1 or below median) and High (Q4 or above median). Each study-specific mean difference and its 95% CIs are represented by squares and horizontal lines, respectively. The diamond represents the pooled mean difference and its 95% CI. The study weights, based on the inverse variance method, are indicated by the size of the squares. Heterogeneity statistics (Chi^2^, I^2^) and tests for the overall effect (Z) are provided to assess the consistency and significance of the findings. SD, standard deviation; IV, inverse variance method for meta-analysis; CI, confidence interval; df, degrees of freedom. (B) The funnel plot with 95% confidence intervals.

Evaluating the association between serum FGF23 levels and kidney function, as measured by eGFR, is crucial in understanding the prognostic value of FGF23 in CKD management. Figure 3A presents the relationship between serum FGF23 levels and eGFR in CKD patients. Seven trials compared eGFR values between participants with low *vs*. high FGF23 levels (Seiler et al., 2010; Isakova et al., 2011b; Kendrick et al., 2011; Ix et al., 2012; Scialla et al., 2014; Isakova et al., 2018; Leidner et al., 2023). Low FGF23 was defined as values in the first quartile (Q1) or below the median, while high FGF23 included values in the fourth quartile (Q4) or above the median.

The pooled result demonstrates a significant inverse relationship between FGF-23 and eGFR. Specifically, the meta-analysis of seven studies found that individuals with elevated serum FGF-23 levels exhibited a mean eGFR that was 17.83 mL/min/1.73m^2^ lower compared to those with lower FGF-23 levels (MD: 17.83, 95% CI [13.23–22.44], I^2^ = 98.6%, *p* = 0.0001) (Fig. 3A). Additionally, the funnel plot does not indicate a potential publication bias (Fig. 3B).

### Elevated FGF-23 levels and risk of kidney failure

**Figure 4:**
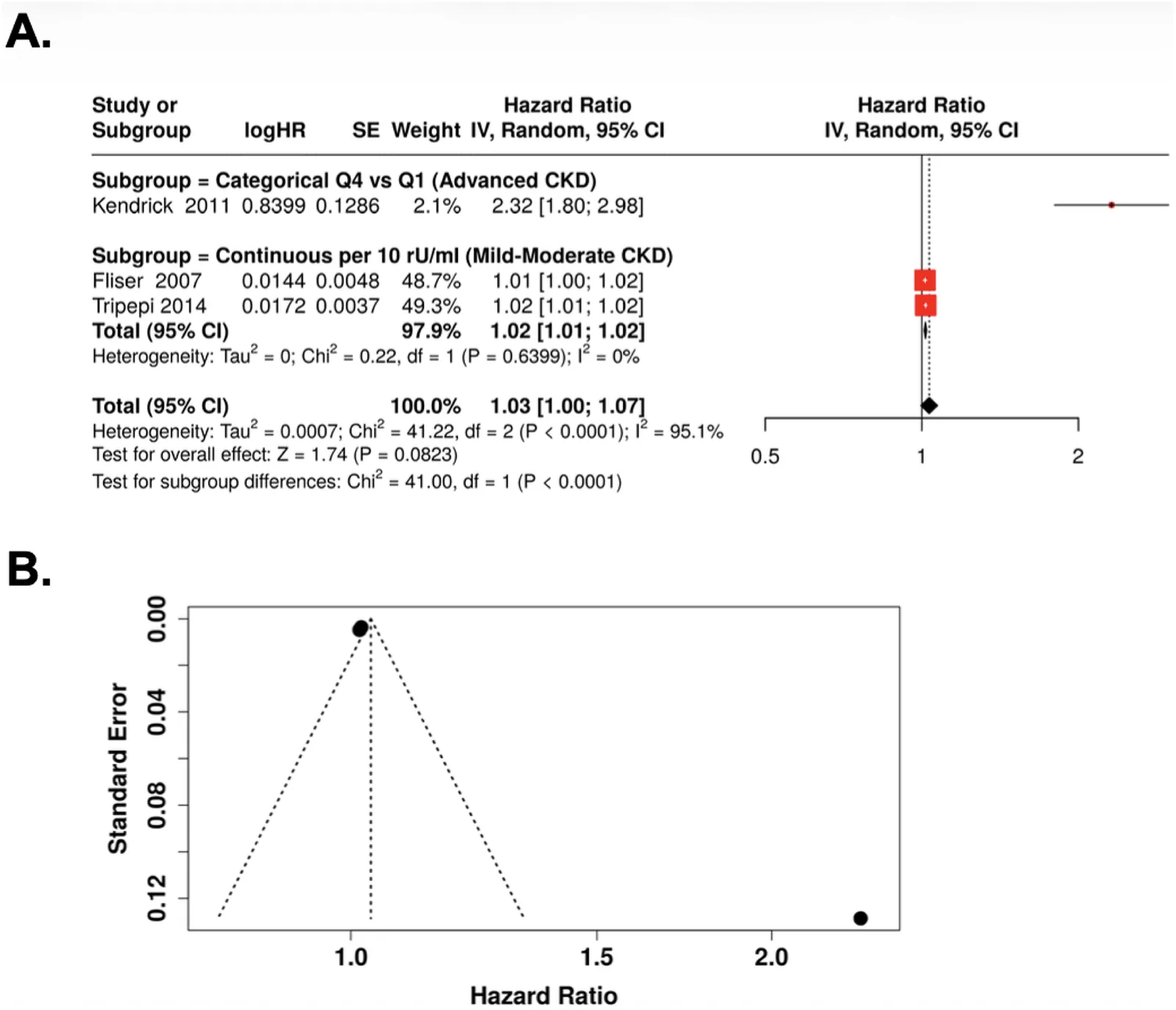
Association between serum FGF23 levels and kidney failure in CKD. (A) The results of a subgroup meta-analysis assessing the association between elevated serum FGF-23 levels and the risk of developing kidney failure in CKD patients. Studies are stratified by exposure assessment type and CKD severity. Each square represents the HR of individual studies, with the size proportional to the study’s weight in the meta-analysis. The horizontal lines represent the 95% CIs. The diamonds indicate the pooled HRs and their 95% CIs for each subgroup. The solid vertical line at 1 represents no association, with values greater than 1 indicating increased risk. Heterogeneity statistics (Chi^2^, I^2^) and tests for the overall effect (Z) are provided to assess the consistency and significance of the findings. (B) The funnel plot with 95% confidence intervals.

Elevated FGF-23 levels have been associated with an increased risk of adverse outcomes, including kidney failure, in CKD patients. Understanding the prognostic value of FGF-23 for CKD progression to kidney failure is essential for improving the management and risk stratification of this patient population. Figure 4A depicts the results of a meta-analysis examining the association between FGF-23 levels and the risk of kidney failure using hazard ratios from Cox regression models. The analysis included data from three prospective cohort studies (Fliser et al., 2007; Tripepi et al., 2015; Kendrick et al., 2011) comprising 1,449 patients with 743 kidney failure events (Fliser et al., 2007; Kendrick et al., 2011; Tripepi et al., 2015).

Given substantial heterogeneity in exposure assessment and study populations, we conducted pre-specified subgroup analyses. In patients with mild-moderate CKD (mean eGFR ~64 ml/min/1.73m^2^), each 10 RU/ml increase in C-terminal FGF-23 was associated with a pooled HR of 1.01 (95% CI [1.01–1.02], I^2^ = 0%, *p* < 0.001), indicating a 1.7% increased risk of kidney failure per unit increment. In advanced CKD, patients in the highest FGF-23 quartile had a 2.32-fold higher risk of dialysis initiation (HR 2.32, 95% CI [1.80–2.98], *p* < 0.001) compared to the lowest quartile (reference group), indicating that high FGF-23 is associated with significantly increased kidney failure risk.

All three studies adjusted for baseline eGFR and other potential confounders, suggesting that FGF-23 provides prognostic information for kidney failure risk beyond that provided by kidney function alone. The funnel plot showed no evidence of publication bias (Fig. 4B). These findings demonstrate that elevated FGF-23 levels are independently associated with progression to kidney failure across the CKD spectrum, with stronger associations observed at higher absolute FGF-23 levels and more advanced disease stages.

### Elevated FGF23 levels associate with increased CVD events

Elevated FGF-23 levels have emerged as a potential risk factor for cardiovascular disease (CVD) outcomes in patients with chronic kidney disease. To investigate this association, we conducted a meta-analysis examining the relationship between FGF-23 levels and CVD events using hazard ratios from Cox proportional hazards models. The analysis included three prospective cohort studies (Ix et al., 2012; Kendrick et al., 2011; Scialla et al., 2014) comprising 6,059 patients with CKD and 502 adjudicated CVD events (Kendrick et al., 2011; Scialla et al., 2014; Alderson et al., 2021).

**Figure 5:**
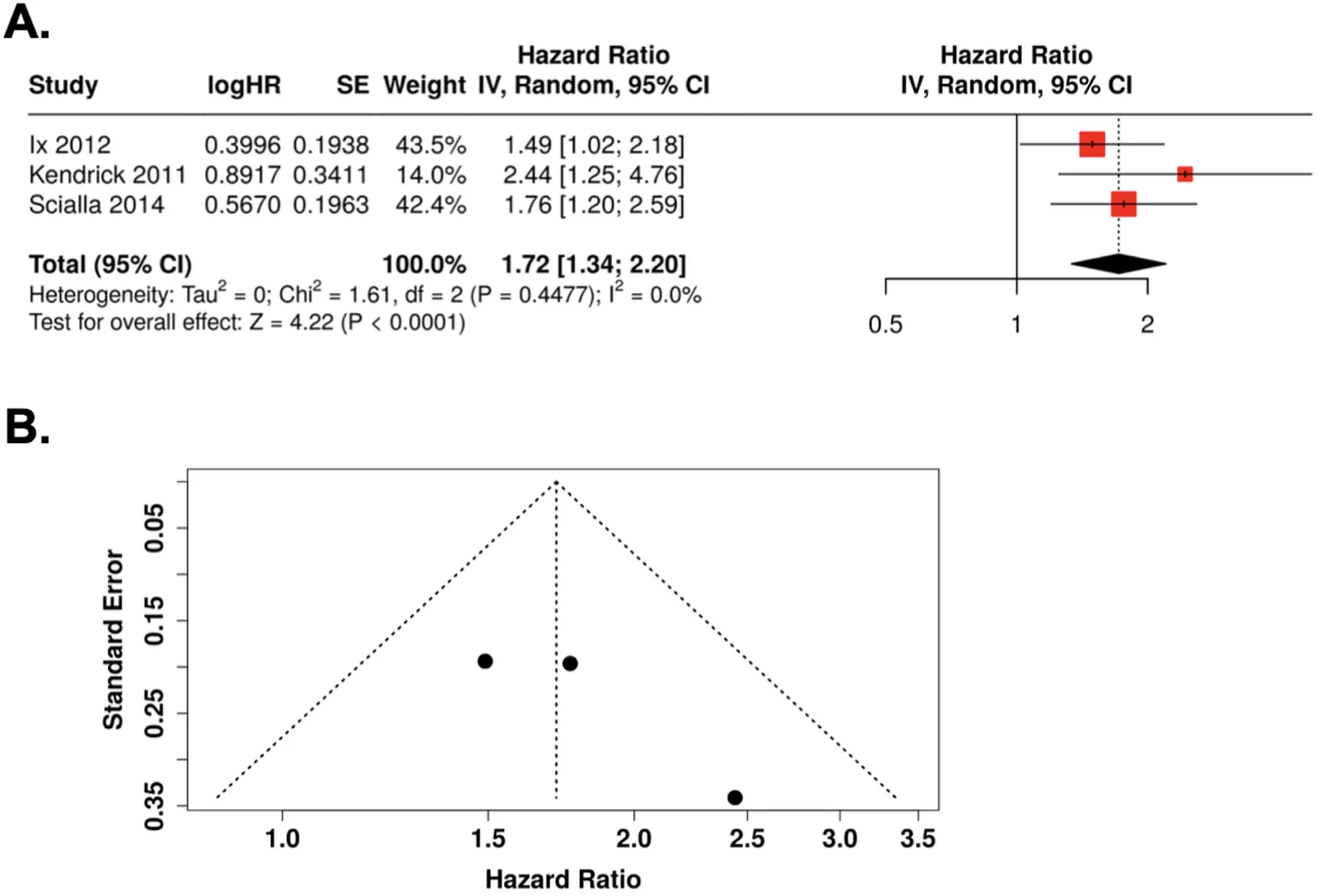
Association between serum FGF23 levels and CVD events in CKD. (A) The results of a meta-analysis examining the association between high and low levels of FGF23 and the risk of CVD events. Each square represents the HR of individual studies, with the size proportional to the study’s weight in the meta-analysis. The horizontal lines represent the 95% CIs. The diamond indicates the pooled HR and its 95% CI. The solid vertical line at 1 represents no difference between high and low FGF23 levels, with values to the left favoring the low FGF23 level (lower risk) and values to the right favoring the high FGF23 level (higher risk). Heterogeneity statistics (Chi^2^, I^2^) and tests for the overall effect (Z) are provided to assess the consistency and significance of the findings. (B) The funnel plot with 95% confidence intervals.

Figure 5A presents the forest plot of this meta-analysis. Patients in the highest FGF-23 quartile demonstrated a significantly increased risk of CVD events compared to those in the lowest quartile (reference group), with a pooled HR of 1.72 (95% CI [1.34–2.20], *p* < 0.0001), indicating a 72% increased risk of cardiovascular events. Notably, the analysis revealed excellent homogeneity across studies (I^2^ = 0%, Chi^2^ = 1.61, *p* = 0.45), indicating highly consistent findings despite differences in study populations and CVD event definitions. All three studies employed comprehensive adjustment for traditional cardiovascular risk factors, kidney function parameters, and mineral metabolism markers, demonstrating that FGF-23 provides independent prognostic information for CVD risk beyond established risk factors. The funnel plot showed no evidence of publication bias (Fig. 5B).

Elevated FGF-23 was associated with a 72% increased risk of CVD events (HR 1.72, 95% CI [1.34–2.20]) in patients with the highest FGF-23 levels.

### Elevated FGF23 levels and risk of heart failure hospitalization

Given the established relationship between elevated FGF-23 levels and increased CVD risk, we examined the specific association between FGF-23 and heart failure (HF) events in chronic kidney disease patients. FGF-23 has been hypothesized to promote cardiac remodeling and left ventricular hypertrophy through direct FGF receptor activation in cardiac myocytes, independent of its phosphaturic effects. To assess this association, we conducted a meta-analysis using hazard ratios from Cox proportional hazards models comparing the risk of HF hospitalization in individuals with the highest *vs*. lowest quartiles of FGF-23 levels. The analysis included three large prospective cohort studies (Ix et al., 2012; Scialla et al., 2014; Leidner et al., 2023) comprising 8,490 patients with chronic kidney disease and 1,352 adjudicated heart failure hospitalizations over extended follow-up periods (median 3.7 to 10.8 years) (Scialla et al., 2014; Leidner et al., 2023; Kidney Disease: Improving Global Outcomes (KDIGO), CKD Work Group, 2024).

**Figure 6:**
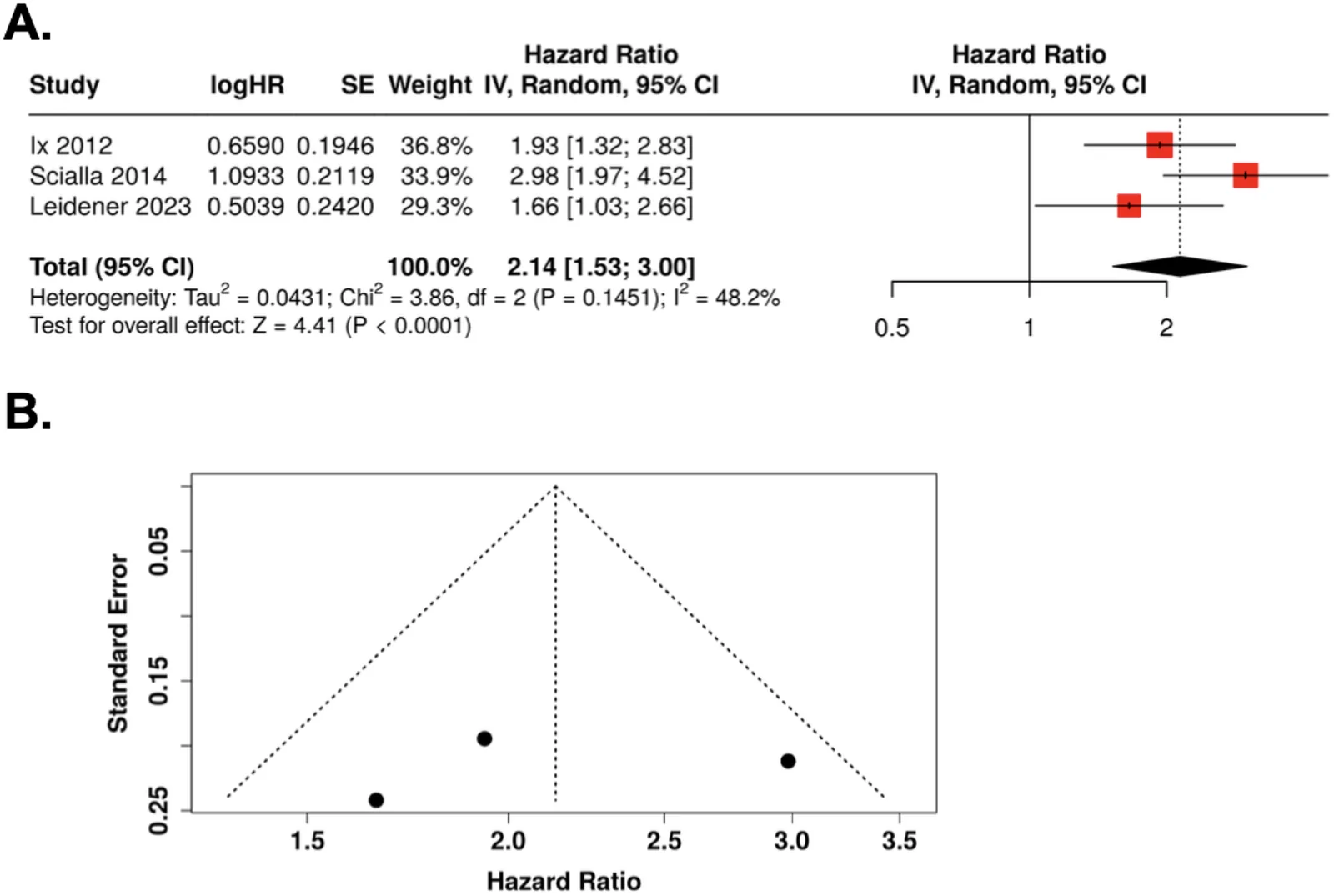
Association between serum FGF23 levels and HF events in CKD. (A) The results of a meta-analysis examining the association between high and low levels of FGF23 and the risk of HF events. Each square represents the HR of individual studies, with the size proportional to the study’s weight in the meta-analysis. The horizontal lines represent the 95% CIs. The diamond indicates the pooled HR and its 95% CI. The solid vertical line at 1 represents no difference between high and low FGF23 levels, with values to the left favoring the low FGF23 level (lower risk) and values to the right favoring the high FGF23 level (higher risk). Heterogeneity statistics (Chi^2^, I^2^) and tests for the overall effect (Z) are provided to assess the consistency and significance of the findings. (B) The funnel plot with 95% confidence intervals.

The pooled HR of 2.14 (95% CI [1.53–3.00], *p* < 0.0001) indicates that patients in the highest FGF-23 quartile had more than twice the risk (114% increased risk) of heart failure hospitalization compared to those in the lowest quartile (reference group) (Fig. 6A). This association was highly statistically significant (Z = 4.41, *p* < 0.0001) and represents the strongest observed association among all cardiovascular outcomes examined. The moderate heterogeneity (I^2^ = 48.2%, *p* = 0.15) is attributable to differences in study populations and methodologies. The heterogeneity may also reflect differences in heart failure definitions, with some studies including all heart failure hospitalizations while others specifically examined incident heart failure or heart failure subtypes. Despite these differences, all three studies consistently demonstrated significant positive associations after comprehensive multivariable adjustment. The funnel plot showed no evidence of publication bias (Fig. 6B).

These findings establish FGF-23 for heart failure (HR 2.14, 95% CI [1.53–3.00]).

### Elevated FGF23 levels associate with increased mortality risk

Given the established associations between elevated FGF23 levels and increased risks of kidney failure and cardiovascular complications in CKD patients, we further examined the relationship between FGF23 and all-cause mortality in this population. Understanding the prognostic value of FGF23 for mortality is crucial, as CKD patients face substantially higher mortality rates compared to the general population.

**Figure 7:**
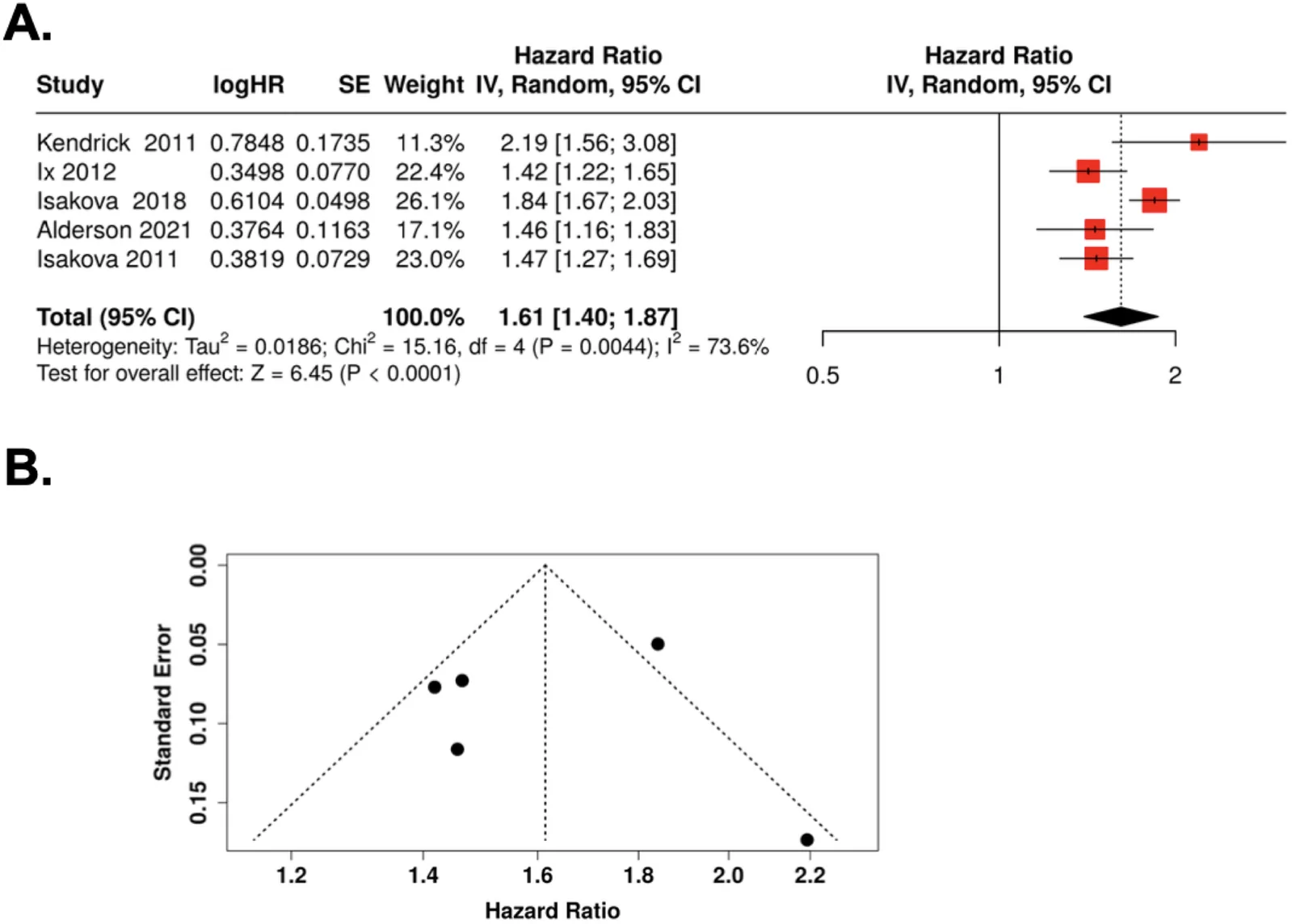
Association between serum FGF23 levels and all-cause mortality in CKD. (A) The results of a meta-analysis examining the association between high and low levels of FGF23 and the risk of all-cause mortality. Each square represents the HR of individual studies, with the size proportional to the study’s weight in the meta-analysis. The horizontal lines represent the 95% CIs. The diamond indicates the pooled HR and its 95% CI. The solid vertical line at 1 represents no difference between high and low FGF23 levels, with values to the left favoring the low FGF23 level (lower risk) and values to the right favoring the high FGF23 level (higher risk). Heterogeneity statistics (Chi^2^, I^2^) and tests for the overall effect (Z) are provided to assess the consistency and significance of the findings. (B) The funnel plot with 95% confidence intervals.

The meta-analysis for this outcome included data from five studies (Alderson et al., 2021; Isakova et al., 2011b, 2018; Ix et al., 2012; Kendrick et al., 2011) comprising 9,998 participants with 3,545 deaths (Isakova et al., 2011b; Kendrick et al., 2011; Ix et al., 2012; Isakova et al., 2018; Alderson et al., 2021). The pooled HR of 1.61 (95% CI [1.40–1.87]) indicates that individuals with high FGF-23 levels had a 61% increased risk of all-cause mortality compared to those with low FGF-23 levels (Fig. 7A), with high statistical significance (Z = 6.45, *p* < 0.0001). Additionally, no potential publication bias is indicated by the funnel plot (Fig. 7B). These results underscore FGF23’s value in predicting heart failure risk in CKD.

## Discussion

### FGF23 as a potential indicator of CKD progression and associated outcomes

Our systematic review and meta-analysis, which includes 12 prospective cohort studies with 21,042 participants, highlights the significant prognostic value of FGF23 in managing pre-dialysis CKD patients. The results demonstrate that elevated FGF23 levels are consistently associated with the deterioration of kidney function, increased risk of kidney failure, CVD events, HF, and all-cause mortality in CKD patients.

Firstly, the analysis of data from multiple prospective cohort studies revealed a clear trend of increasing FGF23 levels as CKD progressed to more advanced stages. This observed association suggests that FGF23 may serve as a valuable biomarker for monitoring the trajectory of kidney function decline in CKD patients. By providing insight into the dynamic changes in FGF23 levels alongside the deterioration of kidney function, these results underscore the potential utility of FGF23 in identifying individuals at increased risk of CKD progression.

Furthermore, the meta-analysis examining the relationship between FGF23 and eGFR demonstrated a significant inverse correlation. Individuals with elevated serum FGF23 levels exhibited a mean eGFR that was nearly 18 mL/min/1.73m^2^ lower compared to those with lower FGF23 levels. This substantial difference of 18 mL/min/1.73m^2^ in eGFR is clinically meaningful, as CKD patients typically experience a gradual decline in kidney function of 2–4 mL/min/1.73m^2^ per year (Russo et al., 2024). This consistent trend across the included studies highlights the association between higher FGF23 and poorer kidney function in the CKD population.

The cross-sectional relationship between FGF-23 and CKD stage or eGFR does not, by itself, establish FGF-23 as a predictor of future CKD progression, as it may partly reflect reduced renal clearance leading to FGF-23 accumulation rather than a primary driver of disease progression. However, longitudinal data from Fliser et al. (2007) and Isakova et al. (2011a) demonstrated that baseline FGF-23 predicted subsequent kidney function decline independent of baseline eGFR, supporting a prognostic role beyond this cross-sectional limitation.

Regarding the risk of adverse outcomes, the meta-analysis found that CKD patients with high FGF23 levels had a significantly higher risk of kidney failure, CVD events, HF, and all-cause mortality compared to those with low FGF23 levels. Specifically, individuals with high FGF-23 levels had a 2.32-fold increased risk of kidney failure, a 1.72-fold increased risk of CVD events, a 2.14-fold increased risk of HF events, and a 1.61-fold increased risk of all-cause mortality. These findings underscore the potential of FGF23 as a prognostic biomarker in identifying CKD patients at increased risk of these critical outcomes.

Collectively, these results suggest that FGF23 may be a valuable indicator of CKD progression and a predictor of adverse outcomes in this patient population. By incorporating FGF23 assessment into clinical practice, healthcare providers may be able to apply targeted interventions and enhanced monitoring for individuals with elevated FGF23 levels, potentially mitigating the risks of kidney failure, cardiovascular complications, and mortality associated with CKD. Further research is warranted to elucidate the underlying mechanisms and explore the clinical applications of FGF23 in the management of chronic kidney disease.

### Comparison with previous meta-analyses on FGF23 in CKD

Our findings align with and extend several previous meta-analyses examining the prognostic value of FGF23 in CKD. Xue et al. (2017) conducted a meta-analysis of 15 studies with 15,355 patients and demonstrated that elevated FGF23 was significantly associated with increased all-cause mortality (risk ratio (RR) 1.37, 95% CI [1.15–1.63]) and cardiovascular mortality (RR 1.37, 95% CI [1.15–1.63]) in CKD patients. Similarly, Marthi et al. (2018) analyzed 17 studies and found elevated FGF23 to be independently associated with greater risks of cardiovascular events and all-cause mortality. Furthermore, unlike Liu et al.’s (2022) JASN meta-analysis which included a broader range of biomarkers, our study focuses specifically on FGF23 and its predictive value for CKD progression and related outcomes.

Our current meta-analysis builds upon these previous works in several important ways. While our findings are consistent with the direction of results reported by Xue et al. (2017) in their 2017 meta-analysis of FGF-23 and all-cause mortality in pre-dialysis CKD patients, our current study substantially expands upon their work in several important ways. First, our analysis encompasses a larger and more diverse cohort (21,042 *vs*. 15,355 participants) and includes more recent studies. Second, while Xue et al. (2017) primarily focused on all-cause mortality with limited cardiovascular outcome data, our study comprehensively examines multiple clinically relevant endpoints including kidney failure, cardiovascular events, heart failure, and mortality, with specific risk quantification for each outcome. Third, our meta-analysis uniquely investigates the relationship between FGF-23 and eGFR across CKD stages, demonstrating a substantial mean difference in kidney function between high and low FGF-23 groups (17.83 mL/min/1.73m^2^). Finally, our study provides a detailed assessment of FGF-23 levels across different CKD stages, offering insights into the potential value of this biomarker for monitoring disease progression. These novel aspects of our analysis provide a more comprehensive understanding of FGF-23’s role in CKD, extending significantly beyond previous meta-analyses in both scope and clinical implications.

A distinguishing feature of our analysis is the systematic evaluation of FGF23 levels across CKD stages, clearly demonstrating the progressive increase in FGF23 as kidney function declines. This graded relationship provides additional support for the potential usefulness of FGF23 as a biomarker for monitoring disease progression. The consistency between our findings and previous meta-analyses strengthens the evidence supporting FGF23 as a valuable prognostic biomarker in CKD. However, by expanding the scope, our study contributes additional insights that may help guide the clinical application of FGF23 testing in CKD management.

While our meta-analysis establishes FGF-23’s prognostic significance in CKD, complementary research has examined therapeutic modalities for FGF-23 reduction. Takkavatakarn et al. (2022) systematically reviewed FGF-23 lowering strategies, demonstrating that non-calcium-based phosphate binders, iron supplements, calcimimetics, and preservation of residual renal function effectively reduce FGF-23 levels in CKD patients (Takkavatakarn et al., 2022). This therapeutic evidence provides crucial context for our prognostic findings.

The distinction between prognostic biomarker validation and therapeutic intervention represents fundamentally different but complementary scientific questions. Our demonstration that elevated FGF-23 levels (highest *vs*. lowest quartile) are associated with a 2.32-fold increased risk of kidney failure (HR 2.32, 95% CI [1.80–2.98]), 1.72-fold increased risk of cardiovascular events (HR 1.72, 95% CI [1.34–2.20]), 2.14-fold increased risk of heart failure (HR 2.14, 95% CI [1.53–3.00]), and 1.61-fold increased risk of all-cause mortality (HR 1.61, 95% CI [1.40–1.87]) provides clinical justification for utilizing the FGF-23 lowering strategies identified by Takkavatakarn et al. (2022). Together, these findings support FGF-23 as both a risk stratification tool and potential therapeutic target, enabling precision medicine approaches where prognostic stratification guides therapeutic selection. Future studies should combine both approaches through interventional trials examining whether FGF-23-guided therapy improves outcomes compared to standard care in high-risk patients.

### Potential prognostic value of FGF23 for predicting CKD onset and progression in the general population

The findings from studies also highlight the potential prognostic value of FGF23 not only in patients with established CKD, but also in the general population prior to CKD onset (De Jong et al., 2021; Takkavatakarn et al., 2022). De Jong et al. (2021) demonstrated that higher levels of FGF23 were associated with an increased risk of developing new-onset CKD, defined as reduced glomerular filtration rate (eGFR < 60 mL/min/1.73m^2^) or elevated urinary albumin excretion (>30 mg/24 h), in a large prospective cohort. This relationship remained even after adjusting for known risk factors for kidney disease, suggesting FGF23 may be an independent predictor of CKD initiation.

Similarly, another study supported these findings, reporting that higher baseline FGF23 levels were predictive of rapid kidney function decline, defined as a 15% decrease in eGFR within the first 4 years, in a community-based healthy population (Chen et al., 2022). Importantly, FGF23 outperformed other established markers, such as eGFR and albuminuria, in its ability to identify individuals at high risk of accelerated CKD progression (Chen et al., 2022). Together, these results suggest that FGF23 could be a valuable biomarker for monitoring CKD progression in patients with the disease and for identifying healthy individuals at risk of developing CKD.

### Heterogeneity and limitations of our study

The robustness of our findings is supported by several methodological strengths. First, our analysis included a large, comprehensive sample spanning all pre-dialysis CKD stages 1 to 5, enhancing the generalizability of our results. Second, the included studies demonstrated high methodological quality as evidenced by NOS scores. Third, the prospective design of included studies minimized selection biases, strengthening the validity of our conclusions.

Our meta-analysis revealed varying degrees of heterogeneity across different outcomes, ranging from excellent homogeneity for cardiovascular events (I^2^ = 0%) to substantial heterogeneity for all-cause mortality (I^2^ = 73.6%). There are several key potential sources of this heterogeneity. First, baseline kidney function emerged as an important moderator, with stronger FGF-23-outcome associations observed in advanced CKD compared to early-stage disease. This gradient likely reflects the dual role of FGF-23 as both a marker of CKD severity and an independent pathophysiological mediator of adverse outcomes. Second, differences in study populations (community-dwelling elderly *vs*. clinic-based CKD cohorts) contributed to heterogeneity, particularly for mortality outcomes, reflecting variations in baseline risk profiles and competing causes of death. Third, heterogeneity in outcome definitions (*e.g*., all heart failure *vs*. specific subtypes) introduced variability in effect estimates. Importantly, despite substantial heterogeneity in some analyses, the direction of effect remained consistent across all studies, with elevated FGF-23 uniformly associated with adverse outcomes. This consistency supports the robustness of FGF-23 as a prognostic biomarker across diverse CKD populations and clinical contexts.

There are several important methodological limitations in our study that warrant consideration. A primary challenge was the heterogeneity in FGF-23 measurement methods across included studies. Most studies (10 of 12) used C-terminal FGF-23 assays, which detect both intact hormone and C-terminal fragments, while one study used intact FGF-23 assays measuring only the full-length, biologically active molecule, and one study measured both forms. These assay types differ not only in their target analytes but also in their units of measurement (RU/mL for cFGF-23 *vs*. pg/mL for iFGF-23) and typical concentration ranges (cFGF-23 values are typically 5–10 fold higher due to inclusion of cleavage fragments).

The impact of assay heterogeneity on our meta-analysis findings deserves careful consideration. Theoretically, the two assay types could yield different prognostic associations if C-terminal fragments have biological activity or if fragment accumulation reflects different pathophysiological processes than intact hormone levels. However, several lines of evidence suggest that assay heterogeneity did not substantially compromise our conclusions. First, our analytical approach used relative comparisons within each study (*e.g*., quartile comparisons, per-SD increases) rather than pooling absolute FGF-23 concentrations, which circumvents the problem of different units and concentration ranges. Second, the limited available evidence comparing assay types suggests similar prognostic value—studies directly measuring both assay types in the same cohorts have generally reported comparable associations with clinical outcomes. Third, in our meta-analysis, the single study using intact FGF-23 showed effect estimates consistent with the range observed in C-terminal studies, with overlapping confidence intervals.

Nonetheless, the predominance of C-terminal assays in our meta-analysis (83% of studies) limits our ability to definitively assess whether assay type modifies prognostic associations. Future comparative studies directly measuring both assay types in the same populations would help clarify this issue. Additionally, the lack of standardized FGF-23 assays and universally accepted reference levels remains an important limitation for clinical implementation. These factors may contribute to the heterogeneity observed in our meta-analyses and impact the comparability of results across different studies and clinical settings.

Future research would benefit from efforts to standardize FGF-23 measurement protocols across clinical studies. Although most included studies adjusted for conventional confounding factors such as age, gender, race, eGFR, and comorbidities, the potential influence of unmeasured confounders cannot be excluded. Additionally, the definitions of CVD and renal outcomes varied among studies, encompassing diverse clinical endpoints that may not be entirely comparable. This heterogeneity in outcome definitions could potentially affect the precision of our risk estimates. Given these limitations, our findings should be interpreted with appropriate caution, and future large-scale studies with standardized FGF23 measurement protocols and outcome definitions are needed to validate these results.

### Exclusion of pediatric studies and its impact on study interpretation

A notable limitation of our study is the exclusion of pediatric CKD studies. CKD in children presents unique challenges and characteristics distinct from adult populations, as evidenced by the Chronic Kidney Disease in Children (CKiD) cohort studies (Wong et al., 2012). Pediatric CKD is characterized by specific comorbidities including growth impairment, neurocognitive developmental issues, and high prevalence of cardiovascular complications such as left ventricular hypertrophy (LVH) and hypertension. The pathophysiological mechanisms and progression patterns of CKD in children may differ from adults, potentially influencing the prognostic value of FGF23 in this population. Additionally, pediatric-specific reference ranges and outcome measures would be necessary for accurate interpretation. Future systematic reviews focusing exclusively on pediatric populations are necessary to establish the prognostic utility of FGF23 in childhood CKD.

### Future directions

Importantly, our heterogeneity analyses identified several areas requiring further investigation. Future studies should employ individual patient data (IPD) meta-analysis to more precisely characterize how baseline kidney function, demographic factors, and comorbidity burden modify the FGF-23-outcome relationship. Such analyses would enable exploration of non-linear associations and identification of clinically relevant FGF-23 thresholds that may vary across patient subgroups. Additionally, standardization of FGF-23 assays and outcome definitions across studies would reduce methodological heterogeneity and improve comparability of findings.

## Conclusion

This systematic review and meta-analysis demonstrates that elevated serum FGF-23 is an independent prognostic biomarker in pre-dialysis CKD patients across stages 1–5. Elevated FGF-23 was significantly associated with increased risks of kidney failure, cardiovascular events, heart failure hospitalization, and all-cause mortality. A substantial inverse association between FGF-23 and eGFR was also confirmed across CKD stages. These findings support integrating FGF-23 measurement into routine CKD care to enable earlier risk stratification, guide targeted clinical interventions, and facilitate more individualized patient management.

## Supplemental Information

10.7717/peerj.21668/supp-1 Supplemental Information 1 Complete search strategies by database.

10.7717/peerj.21668/supp-2 Supplemental Information 2 PRISMA checklist.

10.7717/peerj.21668/supp-3 Supplemental Information 3 Systematic Review and/or Meta-Analysis Rationale. Radiation-induced lung toxicity (RILT) remains a dose-limiting factor in thoracic radiotherapy for lung cancer. While transforming growth factor-beta 1 (TGF-β1) has been investigated as a predictive biomarker for RILT, the evidence remains fragmented and inconsistent, and no previous systematic review has comprehensively synthesized the available data to establish TGF-β1’s discriminative performance, optimal measurement strategies, or clinical utility across diverse patient populations. This systematic review and meta-analysis was conducted to evaluate TGF-β1’s predictive performance for RILT across all available prospective studies, establish whether a TGF-β1 ratio threshold can reliably stratify high-risk and low-risk patients, assess the consistency of predictive value across different measurement timepoints and radiation techniques, and identify critical knowledge gaps requiring future validation. This review is intended for radiation oncologists, medical oncologists, and clinical researchers involved in lung cancer treatment planning, as well as translational researchers, medical physicists, clinical trial investigators, and healthcare policymakers evaluating biomarker-guided radiotherapy strategies. This meta-analysis provides evidence-based guidance for implementing TGF-β1 monitoring to identify high-risk patients and defines critical research priorities for future prospective validation trials.

## Additional Information and Declarations

### Data Availability

The following information was supplied regarding data availability:

This is a Systematic review/Meta-analysis.
