# Supplementary material for: Role of fibroblast growth factor 23 in predicting CKD progression and prognosis: a systematic review and meta-analysis
Source: PeerJ. 2026 Aug 24;14:e21668. doi: 10.7717/peerj.21668 (PMC13525752; doi:10.7717/peerj.21668)
Supplement: Supplementary file 1 [file peerj-14-21668-s002.pdf]

PRISMA

PRISMA 2020 Checklist

Section and Topic	Item #	Checklist item	Location where item is reported
**TITLE**			
Title	1	Identify the report as a systematic review.	Title page: "Role of Fibroblast Growth Factor 23 in Predicting CKD Progression and Prognosis: A Systematic Review and Meta-Analysis"
**ABSTRACT**			
Abstract	2	See the PRISMA 2020 for Abstracts checklist.	Abstract section - structured abstract with Background, Methods, Results, and Conclusions
**INTRODUCTION**			
Rationale	3	Describe the rationale for the review in the context of existing knowledge.	Introduction discusses CKD prevalence, need for prognostic tools, and FGF-23's biological relevance
Objectives	4	Provide an explicit statement of the objective(s) or question(s) the review addresses.	Introduction: This meta-analysis aims to assess the prognostic significance of FGF-23 in predicting the progression of CKD, the incidence of end-stage renal disease (ESRD), cardiovascular events, and all-cause mortality among CKD patients.
**METHODS**			
Eligibility criteria	5	Specify the inclusion and exclusion criteria for the review and how studies were grouped for the syntheses.	Materials and Methods, **Inclusion and Exclusion Criteria** section - detailed inclusion criteria and exclusion criteria.
Information sources	6	Specify all databases, registers, websites, organisations, reference lists and other sources searched or consulted to identify studies. Specify the date when each source was last searched or consulted.	Materials and Methods, **Search Strategy and Study Selection** section - PubMed, Cochrane Library, and SCOPUS searched until January 2025; reference lists of original and review papers examined.
Search strategy	7	Present the full search strategies for all databases, registers and websites, including any filters and limits used.	Materials and Methods, **Search Strategy and Study Selection** section - complete search strategy provided with MeSH terms and keywords
Selection process	8	Specify the methods used to decide whether a study met the inclusion criteria of the review, including how many reviewers screened each record and each report retrieved, whether they worked independently, and if applicable, details of automation tools used in the process.	Materials and Methods, **Data Extraction and Methodological Quality Appraisal** section - two reviewers independently screened; disagreements resolved by third reviewer.
Data collection process	9	Specify the methods used to collect data from reports, including how many reviewers collected data from each report, whether they worked independently, any processes for obtaining or confirming data from study investigators, and if applicable, details of automation tools used in the process.	Materials and Methods, **Data Extraction and Methodological Quality Appraisal** section - two reviewers independently extracted data; disagreements resolved by third reviewer; EndNote X8 used for citation management.
Data items	10a	List and define all outcomes for which data were sought. Specify whether all results that were compatible with each outcome domain in each study were sought (e.g. for all measures, time points, analyses), and if not, the methods used to decide which results to collect.	Materials and Methods, **Outcomes** section - five key outcomes defined: CKD progression rate, incidence of ESRD, cardiovascular events, all-cause mortality, and rate of kidney function decline.

PRISMA

PRISMA 2020 Checklist

Section and Topic	Item #	Checklist item	Location where item is reported
	10b	List and define all other variables for which data were sought (e.g. participant and intervention characteristics, funding sources). Describe any assumptions made about any missing or unclear information.	Materials and Methods, **Data Extraction and Methodological Quality Appraisal** section - variables extracted include first author, study design, publication year, countries, study size, gender, average age, follow-up duration, FGF23 measurement details, CKD stages, kidney functions, and outcome data.
Study risk of bias assessment	11	Specify the methods used to assess risk of bias in the included studies, including details of the tool(s) used, how many reviewers assessed each study and whether they worked independently, and if applicable, details of automation tools used in the process.	Materials and Methods, **Data Extraction and Methodological Quality Appraisal** section - Newcastle-Ottawa Scale (NOS) used; two reviewers independently assessed quality; disagreements resolved by third reviewer.
Effect measures	12	Specify for each outcome the effect measure(s) (e.g. risk ratio, mean difference) used in the synthesis or presentation of results.	Materials and Methods, **Statistical Analysis** section - odds ratios (OR) for categorical outcomes; mean differences (MD) for continuous outcomes; all with 95% confidence intervals.
Synthesis methods	13a	Describe the processes used to decide which studies were eligible for each synthesis (e.g. tabulating the study intervention characteristics and comparing against the planned groups for each synthesis (item #5)).	Materials and Methods, **Selection of Studies** section and Results, **Selection of Studies section** - PRISMA flowchart shows selection process; studies selected based on predefined inclusion criteria.
	13b	Describe any methods required to prepare the data for presentation or synthesis, such as handling of missing summary statistics, or data conversions.	Materials and Methods, **Data Extraction** section - FGF23 assay types documented; subgroup analyses planned based on assay type.
	13c	Describe any methods used to tabulate or visually display results of individual studies and syntheses.	Materials and Methods, **Statistical Analysis** section - forest plots and funnel plots used; Table 1 presents study characteristics.
	13d	Describe any methods used to synthesize results and provide a rationale for the choice(s). If meta-analysis was performed, describe the model(s), method(s) to identify the presence and extent of statistical heterogeneity, and software package(s) used.	Materials and Methods, **Statistical Analysis** and **Heterogeneity** section - RevMan 5.4 and metaanalysisonline.com used; random-effects model selected due to anticipated heterogeneity; I^2 statistics used to assess heterogeneity.
	13e	Describe any methods used to explore possible causes of heterogeneity among study results (e.g. subgroup analysis, meta-regression).	Materials and Methods, **Data Extraction** section - subgroup analyses based on FGF23 assay type (cFGF-23 vs iFGF-23).
	13f	Describe any sensitivity analyses conducted to assess robustness of the synthesized results.	Materials and Methods, **Statistical Analysis** section - both fixed-effects and random-effects models employed for sensitivity analysis; results remained consistent.
Reporting bias assessment	14	Describe any methods used to assess risk of bias due to missing results in a synthesis (arising from reporting biases).	Results section - funnel plots presented for each outcome to assess publication bias (Figures 3B, 4B, 5B, 6B, 7B).
Certainty assessment	15	Describe any methods used to assess certainty (or confidence) in the body of evidence for an outcome.	Not explicitly described - this is a limitation.
**RESULTS**			

PRISMA

PRISMA 2020 Checklist

Section and Topic	Item #	Checklist item	Location where item is reported
Study selection	16a	Describe the results of the search and selection process, from the number of records identified in the search to the number of studies included in the review, ideally using a flow diagram.	Results, **Selection of Studies** section and Figure 1 - PRISMA flowchart shows 1,724 initial articles, 683 duplicates removed, 1,041 excluded during screening, 45 excluded during full-text review, 12 studies included.
	16b	Cite studies that might appear to meet the inclusion criteria, but which were excluded, and explain why they were excluded.	Results, **Selection of Studies** section - states 45 articles excluded during full-text review for not meeting inclusion criteria.
Study characteristics	17	Cite each included study and present its characteristics.	Table 1 - comprehensive characteristics of all 12 included studies; References section lists all included studies.
Risk of bias in studies	18	Present assessments of risk of bias for each included study.	Table 2 - Newcastle-Ottawa Scale scores for all 12 studies; Results, **Critical Appraisal of the Included Studies** section.
Results of individual studies	19	For all outcomes, present, for each study: (a) summary statistics for each group (where appropriate) and (b) an effect estimate and its precision (e.g. confidence/credible interval), ideally using structured tables or plots.	Figures 3A, 4A, 5A, 6A, 7A - forest plots showing individual study results and effect estimates with 95% confidence intervals for each outcome.
Results of syntheses	20a	For each synthesis, briefly summarise the characteristics and risk of bias among contributing studies.	Results sections for each outcome - describes number of studies included, heterogeneity statistics, and study quality.
	20b	Present results of all statistical syntheses conducted. If meta-analysis was done, present for each the summary estimate and its precision (e.g. confidence/credible interval) and measures of statistical heterogeneity. If comparing groups, describe the direction of the effect.	Results sections - pooled results presented for each outcome with odds ratios/mean differences, 95% CIs, and heterogeneity statistics (I^2 values).
	20c	Present results of all investigations of possible causes of heterogeneity among study results.	Results sections - heterogeneity discussed for each outcome; assay type differences acknowledged.
	20d	Present results of all sensitivity analyses conducted to assess the robustness of the synthesized results.	Materials and Methods, **Statistical Analysis** section - mentions sensitivity analyses with both fixed and random effects models; supplementary figures referenced.
Reporting biases	21	Present assessments of risk of bias due to missing results (arising from reporting biases) for each synthesis assessed.	Results sections - funnel plots presented and discussed for each outcome, stating "no potential publication bias indicated".
Certainty of evidence	22	Present assessments of certainty (or confidence) in the body of evidence for each outcome assessed.	Not explicitly presented - this is a limitation.
**DISCUSSION**			
Discussion	23a	Provide a general interpretation of the results in the context of other evidence.	Discussion, **Comparison with Previous Meta-Analyses on FGF23 in CKD** section - compares findings with Xue et al. and Marthi et al. meta-analyses.
	23b	Discuss any limitations of the evidence included in the review.	Discussion, **Heterogeneity and Limitations of Our Study section** - discusses FGF-23 assay heterogeneity, potential unmeasured confounders, outcome

PRISMA

PRISMA 2020 Checklist

Section and Topic	Item #	Checklist item	Location where item is reported
			definition variations, database search limitations, and pediatric study exclusion.
	23c	Discuss any limitations of the review processes used.	Discussion, **Heterogeneity and Limitations of Our Study** section - acknowledges search limited to PubMed/Cochrane, exclusion of Embase.
	23d	Discuss implications of the results for practice, policy, and future research.	Discussion, **Future Directions** section and Conclusions - discusses clinical applications, need for standardized assays, therapeutic targeting, and future research directions.
**OTHER INFORMATION**			
Registration and protocol	24a	Provide registration information for the review, including register name and registration number, or state that the review was not registered.	Materials and Methods, **Search Strategy and Study Selection** section-registered in the International Database of Prospective Systematic Reviews (PROSPERO, Registration No. CRD42024613649).
	24b	Indicate where the review protocol can be accessed, or state that a protocol was not prepared.	PROSPERO registration number provided (CRD42024613649).
	24c	Describe and explain any amendments to information provided at registration or in the protocol.	Not described
Support	25	Describe sources of financial or non-financial support for the review, and the role of the funders or sponsors in the review.	Declarations, Funding - Not Applicable.
Competing interests	26	Declare any competing interests of review authors.	Declarations, Competing Interest - Jia-Wei Hsu, Hsiang-Ping Wang, and Tsay-I Chiang declare that they have no conflict of interests.
Availability of data, code and other materials	27	Report which of the following are publicly available and where they can be found: template data collection forms; data extracted from included studies; data used for all analyses; analytic code; any other materials used in the review.	Declarations, Availability of data and materials - The datasets used and/or analyzed during the current study are available from the corresponding author on reasonable request.

From: Page MJ, McKenzie JE, Bossuyt PM, Boutron I, Hoffmann TC, Mulrow CD, et al. The PRISMA 2020 statement: an updated guideline for reporting systematic reviews. BMJ 2021;372:n71. doi: 10.1136/bmj.n71.
This work is licensed under CC BY 4.0. To view a copy of this license, visit https://creativecommons.org/licenses/by/4.0/
