# Supplementary material for: Role of fibroblast growth factor 23 in predicting CKD progression and prognosis: a systematic review and meta-analysis
Source: PeerJ. 2026 Aug 24;14:e21668. doi: 10.7717/peerj.21668 (PMC13525752; doi:10.7717/peerj.21668)
Supplement: Supplementary file 2 [file peerj-14-21668-s003.pdf]

Systematic Review and/or Meta-Analysis Rationale

Radiation-induced lung toxicity (RILT) remains a dose-limiting factor in thoracic radiotherapy for lung cancer. While transforming growth factor-beta 1 (TGF-β1) has been investigated as a predictive biomarker for RILT, the evidence remains fragmented and inconsistent, and no previous systematic review has comprehensively synthesized the available data to establish TGF-β1's discriminative performance, optimal measurement strategies, or clinical utility across diverse patient populations. This systematic review and meta-analysis was conducted to evaluate TGF-β1's predictive performance for RILT across all available prospective studies, establish whether a TGF-β1 ratio threshold can reliably stratify high-risk and low-risk patients, assess the consistency of predictive value across different measurement timepoints and radiation techniques, and identify critical knowledge gaps requiring future validation. This review is intended for radiation oncologists, medical oncologists, and clinical researchers involved in lung cancer treatment planning, as well as translational researchers, medical physicists, clinical trial investigators, and healthcare policymakers evaluating biomarker-guided radiotherapy strategies. This meta-analysis provides evidence-based guidance for implementing TGF-β1 monitoring to identify high-risk patients and defines critical research priorities for future prospective validation trials.
