# Supplementary material for: Role of fibroblast growth factor 23 in predicting CKD progression and prognosis: a systematic review and meta-analysis
Source: PeerJ. 2026 Aug 24;14:e21668. doi: 10.7717/peerj.21668 (PMC13525752; doi:10.7717/peerj.21668)
Supplement: Supplementary file 3 [file peerj-14-21668-s001.docx]

**Supplementary Table S1. Complete search strategies by database.**

| Database | Search Strategy |
| --- | --- |
| PubMed | ((“Renal Insufficiency, Chronic”[Mesh] OR “chronic kidney disease”[Title/Abstract] OR “CKD”[Title/Abstract] OR “chronic renal insufficiency”[Title/Abstract] OR “chronic kidney failure”[Title/Abstract])) AND (“Fibroblast Growth Factor 23”[Mesh] OR “FGF-23”[Title/Abstract] OR “FGF23”[Title/Abstract] OR “fibroblast growth factor 23”[Title/Abstract])) AND (“Disease Progression”[Mesh] OR “Kidney Failure, Chronic”[Mesh] OR “Cardiovascular Diseases”[Mesh] OR “Bone Diseases, Metabolic”[Mesh] OR “Mortality”[Mesh] OR “Glomerular Filtration Rate”[Mesh] OR “ESRD”[Title/Abstract] OR “end stage renal disease”[Title/Abstract] OR “cardiovascular event*”[Title/Abstract] OR "bone mineral disorder*”[Title/Abstract] OR “all-cause mortality”[Title/Abstract] OR “eGFR”[Title/Abstract] OR “estimated glomerular filtration rate”[Title/Abstract]) |
| Cochrane Library | ([mh “Renal Insufficiency, Chronic”] OR “chronic kidney disease” OR “CKD” OR “chronic renal insufficiency” OR “chronic kidney failure”) AND ([mh “Fibroblast Growth Factor 23”] OR “FGF-23” OR “FGF23” OR “fibroblast growth factor 23”) AND ([mh “Kidney Failure, Chronic”] OR [mh “Disease Progression”] OR [mh “Cardiovascular Diseases”] OR [mh “Bone Diseases, Metabolic”] OR [mh “Mortality”] OR [mh “Glomerular Filtration Rate”] OR “ESRD” OR “end stage renal disease” OR “cardiovascular event*” OR “bone mineral disorder*” OR “all-cause mortality” OR “eGFR” OR “estimated glomerular filtration rate”) |
| Embase | The search was conducted using equivalent keyword terms; the database-specific string was not separately retained. |
| SCOPUS |  |
